# (Over)eating out at major UK restaurant chains: observational study of energy content of main meals

**DOI:** 10.1136/bmj.k4982

**Published:** 2018-12-12

**Authors:** Eric Robinson, Andrew Jones, Victoria Whitelock, Bethan R Mead, Ashleigh Haynes

**Affiliations:** Institute of Psychology, Health & Society, University of Liverpool, Liverpool L69 7ZA, UK

## Abstract

**Objectives:**

To examine the energy content of main meals served in major UK restaurant chains and compare the energy content of meals in fast food and “full service” restaurant chains.

**Design:**

Observational study.

**Setting:**

Menu and nutritional information provided by major UK restaurant chains.

**Main outcome measures:**

Mean energy content of meals, proportion of meals meeting public health recommendations for energy consumption (≤600 kcal), and proportion of meals with excessive energy content (≥1000 kcal).

**Results:**

Main meals from 27 restaurant chains (21 full service; 6 fast food) were sampled. The mean energy content of all eligible restaurant meals (13 396 in total) was 977 (95% confidence interval 973 to 983) kcal. The percentage of all meals that met public health recommendations for energy content was low (9%; n=1226) and smaller than the percentage of meals with an excessive energy content (47%; 6251). Compared with fast food restaurants, full service restaurants offered significantly more excessively calorific main meals, fewer main meals meeting public health recommendations, and on average 268 (103 to 433) kcal more in main meals.

**Conclusions:**

The energy content of a large number of main meals in major UK restaurant chains is excessive, and only a minority meet public health recommendations. Although the poor nutritional quality of fast food meals has been well documented, the energy content of full service restaurant meals in the UK tends to be higher and is a cause for concern.

**Registration:**

Study protocol and analysis strategy pre-registered on Open Science Framework (https://osf.io/w5h8q/).

## Introduction

The prevalence of overweight and obesity has increased markedly across most of the developed world.^1^ Increases in energy intake caused by major changes to the food environment have been identified as a key factor explaining weight gain at the population level.^2^ ^3^ ^4^ In the UK, meals are regularly consumed out of the home; data collected from 2008-12 showed that a quarter of UK adults ate out once a week or more often.^5^ However, a more recent report from the UK Food Standards Agency in 2016 indicates that eating out of the home may be becoming more common, with 39% of UK adults reporting eating out at least once a week.^6^ Several studies suggest that people who eat out of the home more often are at increased risk of weight gain and obesity.^7^ Fast food restaurants in particular have been highlighted as providing meals that are low in nutritional quality.^8^ ^9^ Some evidence also suggests that a higher geographical density of fast food restaurants is associated with an increased risk of obesity.^10^ ^11^ Because of this, public health calls have been made to limit where fast food restaurant outlets can operate in the UK.^12^ ^13^ However, more traditional “full service” restaurants also contribute substantially to the out of home dining market in the UK.^14^

Recent public health recommendations made by Public Health England suggest that adults should aim to consume 600 kcal or less for their main lunch and dinner meals to avoid excess daily energy intake and maintain a healthy body weight.^15^ This is in part motivated by Public Health England’s estimate that the average adult in the UK is consuming an excess of 195 kcal a day.^15^ Because the amount of energy a person consumes during a meal is strongly influenced by the energy density and portion size of the food served,^16^ ^17^ ^18^ ^19^ meals provided to consumers that are high in energy promote excess energy intake and are problematic for public health. However, public health action on improving the nutritional quality of food prepared outside of the home has to date focused largely on encouraging the food industry to make reductions to the energy content of supermarket food,^20^ rather than focusing on the restaurant sector. To date, the number of kilocalories in main meals served by major UK restaurant chains has not been examined, so whether consumers can adhere to public health recommendations for meal energy consumption when eating in these establishments is unclear. Moreover, legislation has been passed that will result in kilocalorie labelling of all food products sold by major chain restaurants becoming mandatory in the US.^21^ Similar legislation is being considered by the UK government, but mandatory labelling will come at a financial cost to the food industry, which may cause challenges to legislation, as was the case in the US.^22^ To overcome such challenges, it will be important to understand the extent to which major UK restaurant chains are contributing to overconsumption by examining the typical energy content of main meals and the availability of main meals meeting public health recommendations for energy consumption.

We examined the energy content of main meals (lunch and dinner) sold by major restaurant chains in the UK. We also compared the energy content of main meals in fast food and traditional full service restaurant chains. We reasoned that this comparison would be important for several reasons. Firstly, although the energy content of full service restaurant meals has received little attention in the UK, a few studies of North American dining suggest that the energy content for these restaurant types can be excessive.^23^ Secondly, we speculate that over time the negative publicity about the poor nutritional quality of fast food may have caused this sector to provide meal options with lower energy content on their menus, reformulate existing meals, or both,^24^ whereas the full service restaurant sector has presumably not faced this pressure. Thus, we hypothesised that, somewhat counterintuitively, the energy content of main meals in full service restaurant chains would be more excessive than that of fast food restaurant chains in the UK.

## Methods

### Restaurant sampling and characterisation

As our aim was to examine major UK restaurant chains, we included all chains with 50 or more outlets in the UK. We were aware of no formal classification of what determines a “major” restaurant chain. We chose 50 outlets or more as this allowed us to include all restaurant chains that were consistently high in annual turnover and popularity according to market reports we accessed. Our scoping research also indicated that chains with fewer than 50 outlets were less likely to provide online nutritional information for menu items. To identify major restaurant chains, we consulted market reports listing restaurants with the largest number of UK outlets and market research ranking UK restaurant chains by annual turnover, popularity, number of users, and numbers of outlets.^14^ ^25^ ^26^ ^27^ To confirm eligibility, during March-April 2018 one researcher accessed the UK website of each restaurant chain to identify those with at least 50 outlets, and this was independently verified by another researcher. If the number of UK outlets was not provided on a restaurant’s website, we requested this information by email. The supplementary table gives a full list of all restaurant chains identified with at least 50 outlets and the number of outlets per chain.

To categorise restaurant chains as fast food or full service, on the basis of previous research,^28^ we used the following definition of fast food restaurants: “Restaurants that primarily provide consumers with largely pre-prepared ‘quick’ meals with little or no table service, with in-store seating and in which take-away orders are likely to account for a significant proportion of orders.” We did not include coffee shop chains or chains that only provided take away food (that is, no physical restaurant). Two researchers independently coded each eligible restaurant as fast food or full service, and any disagreements were resolved through discussion.

### Data sources and identification of main meal menu options

During April-July 2018 we accessed the UK websites of all eligible restaurant chains and identified current menus and nutritional information. We contacted restaurant chains that did not provide nutritional information on their UK website requested this information.

We aimed to examine the energy content of all “main meal” menu options. We defined a main meal as being a menu option that would normally be the primary dish in a lunch or dinner meal and typically be found in the “main course” part of a restaurant’s menu. Examples of main meal items according to this definition include burger and chips, chicken Caesar salad, spaghetti Bolognese, and jacket potato with a filling. We did not include individually sold food items (such as sides) or sharing menu options (such as tapas), as what combination of individual items or sharing menu options would constitute a main meal was unclear. We did not include starters and dessert menu options, as they are not typically consumed as a main meal. We did not include breakfast menu options, as during a scoping exercise we found that a large number of eligible restaurants did not offer breakfast menu options. We included main meals that could be purchased by any member of the general public, and menu options for specialist consumer groups were ineligible (for example, pensioners’ menu, children’s menu). To minimise seasonal effects, we included only main meal options that seemed to be available all year round. In instances in which a main meal menu option could be customised at the explicit request of the customer (for example, swap default side dish for a different side dish), we selected the default composition of the meal. When a meal menu option required a customer to make an explicit choice (for example, choice of salad or fries), we extracted all possible configurations of the meal and recorded each variant as an individual meal.

During May-June 2018 two researchers independently accessed each restaurant menu and identified eligible meal menu options. See supplementary materials for more detailed information on the coding instructions used by researchers. Discrepancies were resolved by discussion and, if necessary, a third researcher. If a restaurant provided only information on individual food items on their online menu, with no information on which combinations of items constituted a meal option, two researchers visited a local outlet (Liverpool city centre) of that restaurant and recorded eligible menu meal options.

### Extraction of meal energy content

Although nutritional information tends not to be displayed on UK restaurant menus in store, some restaurant chains provide this information on their websites. A researcher accessed online nutritional information for each restaurant and extracted the number of kilocalories for each eligible meal. A second researcher checked the extraction for accuracy. If information on energy content was missing from a restaurant’s nutritional information, we attempted to locate it from elsewhere on the website; if we were not able to do this, we used information from a close to identical meal option (for example, sandwich with brown versus wholegrain bread), if available. Because drinks were not routinely provided with meals in restaurants, we did not include drinks when extracting information on energy content for any meal options that included a drink.

### Inter-coder consistency

We examined the percentage agreement (that is, the proportion of restaurants identified by both researchers as fast food or full service) as an indicator of inter-coder consistency for classification of restaurant type. We had planned to adopt a similar approach for inter-coder consistency of identification of eligible main meal options. However, the very large number of menu items that were clearly not eligible (for example, starters, sides, desserts, drinks, children’s menu) meant that coders did not record a classification (eligible versus not eligible) for every menu item and recorded eligible menu options only. We therefore approximated inter-coder consistency by examining the number of menu options identified that were deemed eligible by both coders independently compared with the number of menu options identified by one of the coders but not the other.

### Planned analyses

To estimate the mean energy content of meal options, we used multilevel modelling, with individual meal options nested within restaurants and restaurants categorised as being fast food or full service. We examined model fit by comparing the log-likelihood ratio statistic (log-likelihood of the multilevel model minus log-likelihood of a single level model) with a χ^2^ distribution with one degree of freedom. We used bootstrapping (500 samples), as this improves the accuracy of parameter values and reduces bias in parameter estimates. Because any difference in meal energy content between fast food and full service restaurants may be in part explained by the two types of restaurant serving different types of meals, we also planned to repeat our analyses for any individual meal types that were provided by the majority of both fast food and full service restaurant chains. We identified two meal types (burger and fries/chips meals, salad meals) that met this requirement and used the same multilevel modelling approach described above.

We also examined the proportion of meals that met UK public health guidelines for recommended energy consumption (≤600 kcal) for a main meal,^15^ as well as the proportion of all meals that were excessively high in energy. We defined “excessive” as meals with an energy content of 1000 kcal or more, as this single meal constitutes 50% and 40% of the recommended total number of daily kilocalories for women (2000 kcal) and men (2500 kcal), respectively, and also constitutes most of the kilocalories that the UK National Health Service recommends a man or women attempting weight loss to eat in a day.^29^ To examine whether fast food and full service restaurants differed in the proportion of main meals that were 600 kcal or less and 1000 kcal or more, we did two multilevel logistic regressions. We used first order marginal quasi-likelihood models. To examine whether two level models (meals within restaurants) were more appropriate than single level models, we examined whether residual variance at the restaurant level was significantly different from 0, by calculating the Wald statistic (variance/standard error)^2^ and comparing this with a χ^2^ distribution with one degree of freedom. We planned in the pre-registered protocol to examine the above with χ^2^ only, and for completeness we report these results in the supplementary material. In all analyses, α was set at 0.05. We used MLWiN v.3 (2017) for multilevel analyses and SPSS 22 for all other analyses.

### Patient and public involvement

No patients or members of the public were directly involved in this study. There are no plans to involve patients or the public in the dissemination of results.

## Results

### Restaurants

We identified 52 restaurant chains with 50 or more outlets in the UK. Of these, we were able to access menu and nutritional information for 30 restaurants and requested this from the remaining chains, one of which provided information. Four of these 31 restaurants did not have items on their menus that constituted meals, selling only individual food items (such as individual pieces of chicken or sushi), and so were not eligible for inclusion. Inter-coder consistency for classification of eligible restaurants as fast food or full service was high (96%; 26/27 restaurants), and the one discrepancy was resolved by discussion between the two coders after accessing the chain’s website. The final number of eligible restaurant chains was 27 (6 fast food, 21 full service; table 1).

**Table 1:** Energy content of meals from eligible restaurant chains included in analyses

Restaurant chain	No of meals	Mean (SD) kcal/meal	No (%) meals ≤600 kcal	No (%) meals ≥1000 kcal
Fast food restaurants (n=6)*:		751 (128)	-	-
Burger King	50	711 (214)	17 (34)	4 (8)
KFC	106	987 (273)	5 (5)	53 (50)
Leon	14	597 (86)	8 (57)	0 (0)
McDonalds	127	726 (242)	35 (28)	14 (11)
Subway†	2436	763 (252)	760 (31)	490 (20)
Wimpy	64	721 (221)	17 (27)	6 (9)
Full service restaurants (n=21)*:		1033 (175)	-	-
All Bar One	33	871 (263)	5 (15)	11 (33)
Ask	44	790 (184)	7 (16)	7 (16)
Bills	16	966 (310)	2 (13)	7 (44)
Chef and Brewer	95	1177 (390)	6 (6)	63 (66)
Ember Inns	75	1085 (334)	5 (7)	45 (60)
Flaming Grill	52	1232 (496)	6 (12)	36 (69)
Harvester	62	1166 (370)	5 (8)	43 (69)
Hungry Horse	333	1358 (472)	19 (6)	261 (78)
JD Wetherspoons	114	1119 (428)	16 (14)	72 (63)
Nando’s†	9293	1019 (231)	282 (3)	4911 (53)
Old English Inns	67	1125 (392)	6 (9)	45 (67)
Pizza Express	34	854 (234)	6 (18)	7 (21)
Pizza Hut	33	975 (238)	4 (12)	19 (58)
Sizzling Pubs	87	1269 (575)	7 (8)	56 (64)
Slug and Lettuce	37	963 (243)	2 (5)	15 (41)
Stone House	23	1275 (323)	0 (0)	18 (78)
Table Table	57	869 (273)	9 (16)	17 (30)
Toby Carvery	20	942 (166)	1 (5)	8 (40)
Vintage Inns	40	1064 (414)	6 (15)	21 (53)
Wagamama	40	836 (259)	7 (18)	12 (30)
Zizzi	44	735 (337)	23 (52)	10 (23)

* For descriptive purposes, values in this row represent mean (SD) of individual restaurant values for mean kcal per meal.

† The relatively large number of eligible meals identified in some restaurant chains was due to a large number of meal variants (eg, chicken with choice of any two sides, sandwich meal with choice of bread type, size, and sides) in these restaurants.

### Meals

Of the meals identified by the first coder (13 422 meals), 99% were also identified by the second coder, and of the meals identified by the second coder (13 444 meals), 99% were identified by the first coder, indicating consistency between the two coders when identifying eligible meals. After discrepancies between the two coders (meals identified by one coder only) were resolved, the final number of eligible meals was 13 507. The large number of eligible meals was mainly attributable to the relatively large number of meals contributed by two restaurant chains offering meals with multiple variants (for example, chicken with a choice of any two sides, sandwich meal with a choice of multiple sides). With these restaurant chains removed, inter-coder consistency as described above remained high (90% and 90%).

### Energy content of meals

Of the 13 507 eligible meals identified, we were able to extract information on energy content for 13 396 (99%) meals. We treated the remaining meals as missing data and did not include them in analyses. Table 1 gives the number of eligible meals per restaurant and raw energy content data per meal for each restaurant.

Across all meals, the average energy content per meal was 977 (95% confidence interval 973 to 983; SE 2) kcal. A two level model structure (meals within restaurants) was a better fit of the data than a single level structure (χ^2^ (df=1) 2918; P<0 .001), indicating that multilevel modelling was appropriate. The variance partition coefficient—the total residual variance that is attributable to restaurants rather than individual meals—was 37%. Type of restaurant (full service versus fast food) was a significant predictor (β=268 (SE 84); 95% confidence interval 103 to 433; P<0.001), explaining 36% of variance at the restaurant level. These results indicate that meals from full service restaurants had 268 kcal more energy than did meals from fast food restaurants, on average.

Across burger and fries/chips meals (n=1904; 1010 full service, 894 fast food), the average energy content was 1171 (SE 7) kcal. The weighted multilevel model showed that a two level structure was a better fit than a single level structure (χ^2^ (df=1) 411; P<0.001), and the variance partition coefficient was 68%. Type of restaurant (full service versus fast food) was a significant predictor (β=414 (SE 141); 138 to 691l; P<0.001), explaining 29% of variance at the restaurant level and indicating that burger meals in full service restaurants had 414 kcal more energy than those in fast food restaurants, on average. Across salad meals (n=304; 92 full service, 212 fast food), the average energy content was 446 (SE 10). A two level structure was a better fit than a single level structure (χ^2^ (df=1) 885; P<0.001, and the variance partition coefficient was 69%. Type of restaurant (full service versus fast food) explained 8% of variance at the restaurant level, and salad meals in full service restaurants had on average 142 kcal more than fast food salad meals, although restaurant type was not a statistically significant predictor in the model (β=142 (SE 99); –52 to 336; P=0.076. Table 2 gives average energy content for burger and salad meals by restaurant.

**Table 2:** Energy content of burger and fries/chips meals and salad meals from eligible restaurant chains included in analyses

Restaurant chain	No of salad meals	Mean (SD) kcal of salad meals	No of burger meals	Mean (SD) kcal of burger meals
Fast food restaurants*:		411 (175); n=6		967 (171); n=4
Burger King	1	210†	24	843 (214)
KFC	3	663 (121)	14	1220 (322)
Leon	4	555 (40)	0	-
McDonalds	8	248 (77)	24	907 (141)
Subway‡	192	416 (124)	0	-
Wimpy	4	372 (68)	22	898 (138)
Full service restaurants*:		559 (261); n=20		1362 (249); n=14
All Bar One	4	606 (295)	6	1055 (247)
Ask	4	650 (268)	0	-
Bills	1	902†	3	1206 (148)
Chef and Brewer	7	558 (191)	8	1459 (188)
Ember Inns	3	575 (175)	7	1295 (294)
Flaming Grill	1	325†	8	1431 (225)
Harvester	4	552 (13)	7	1414 (149)
Hungry Horse	4	394 (296)	14	1966 (771)
JD Wetherspoons	5	494 (139)	14	1565 (318)
Nando’s‡	30	428 (150)	912	1161 (154)
Old English Inns	1	247†	6	1543 (213)
Pizza Express	4	886 (296)	0	-
Pizza Hut	0	-	0	-
Sizzling Pubs	2	280 (103)	10	1521 (401)
Slug and Lettuce	4	736 (215)	5	1280 (149)
Stone House	2	1353 (158)	0	-
Table Table	3	329 (88)	6	1105 (166)
Toby Carvery	2	614 (100)	0	-
Vintage Inns	3	380 (335)	2	1069 (195)
Wagamama	4	414 (48)	0	-
Zizzi	4	467 (213)	0	-

* For descriptive purposes, values in this row represent the mean (SD) kcal of individual restaurant values for salad meals and burger meals.

† No SD as only one eligible meal from restaurant.

‡ The relatively large number of eligible meals identified in some restaurant chains was due to a large number of meal variants (eg, burger meal with choice of multiple sides) in these restaurants.

Because an unexpectedly large amount of variability existed in the number of meals that individual restaurants contributed to the analyses for all meals and specific meal types, we also did weighted multilevel analyses (see supplementary materials).

Of the 13 396 possible meals identified, 1226 (9%) met UK public health recommendations of 600 kcal or less. The total number of meals that contained 1000 kcal or more was 6251 (47%) (see table 1). Logistic models examining proportion of meals of 600 kcal or less showed significant variance at the restaurant level (Wald test statistic=9.0; P=0.002), suggesting that a two level model was appropriate. The odds ratio for restaurant type was 3.2 (95% confidence interval 1.4 to 7.4; P=0.003), suggesting that fast food restaurants were approximately three times more likely to offer meals that contained 600 kcal or less than full service restaurants. The proportion of meals that contained 1000 kcal or more showed significant variance at the restaurant level (Wald test statistic=6.0; P=0.014), suggesting that a two level model was appropriate. The odds ratio for restaurant type was 5.1 (1.7 to 15.0; P=0.002), suggesting that full service restaurants were approximately five times more likely to offer meals of 1000 kcal or more than fast food restaurants

## Discussion

Across the meals from major UK chain restaurants included, the mean energy content of main meals was 977 kcal, a sizeable proportion (47%) were “excessive” in energy content (≥1000 kcal), and only a small minority (9%) were in line with public health recommendations for main meal energy consumption (≤600 kcal). On average, the energy content of main meals served by full service restaurants was 268 kcal higher than that of main meals served by fast food restaurants. Full service restaurants also tended to serve more highly calorific main meals and provide fewer main meals meeting public health recommendations for energy consumption.

### Strengths and weakness of study

We were able to sample a large number of restaurant chains and main meals. However, our analyses were limited to restaurants that provided nutritional information and sold products consistent with our inclusion criteria (27/52 identified chains). This is a weakness of the study, as main meals in restaurants not providing online nutritional information may differ in energy content from those that do. Reliance on self reported information on energy content from restaurant chains is another weakness of the study, and objectively calculated energy content (using laboratory methods) would have been preferable but was not feasible. However, previous research suggests that commercially provided nutritional information tends to be accurate but may underestimate the energy content of some products.^30^ ^31^ Our findings are therefore more likely to underestimate than overestimate energy content of main meals, which means that the energy content of UK restaurant food may be more problematic than our data suggest. Our focus here was on the energy content of main meals. Although energy intake is of most relevance to body weight and obesity at the population level, other aspects of diet (such as salt or saturated fat) also shape health and disease. For example, the amount of salt in most UK supermarket ready meal products does not meet nutritional guidelines,^32^ and the salt content of main meals in UK restaurant may be similarly high. In this study, we did not include larger main meal items that are typically shared by consumers owing to uncertainty about what would constitute one portion. In addition, we examined the number of kilocalories served, and this does not permit us to make conclusions about consumption. Although consumers will not always finish all of a meal served, “plate clearing” is a fairly common behaviour.^33^ ^34^ Because some customers will order a main meal as well as a drink, starter, and/or dessert, we assume that on average the number of kilocalories consumed in both full service and fast food restaurants will be higher still.

### Strengths and weaknesses in relation to other studies

Although research has examined the nutritional quality of restaurant food, this has tended to be done in North America.^23^ ^35^ This is the first study we are aware of to characterise the energy content of main meals served in UK chain restaurants. A limitation of our study was that we did not examine smaller chains or independent restaurants, although both chain and non-chain US restaurants serve excess amounts of energy.^35^ Previous studies examining nutritional quality of other food products (such as supermarket food) have made use of World Health Organization guidelines to examine the proportion of products meeting specific nutrient recommendations (such as energy from saturated fat).^32^ No international guidelines make recommendations about energy consumption per meal. We therefore examined the proportion of main meals meeting Public Health England’s recommendation of 600 kcal or less per meal (lunch and dinner). We also quantified the proportion of main meals that not only failed to meet this recommendation but could be considered to be “excessive” in energy content. Given the lack of consensus on what is an excessive energy content, we defined this as main meals that had 1000 kcal or more, as in a single course this would constitute 50% and 40% of the recommended total number of daily kilocalories for women and men respectively or, viewed in another way, most of the kilocalories that a man or women attempting weight loss is recommended to eat in a day. Although the amount of energy that any person needs to maintain a healthy body weight varies, few people are likely to need more than 1000 kcal from a single main meal to maintain a healthy body weight. By using 1000 kcal or more as a threshold for an excessive energy content in this study, we are not suggesting that this should become a default threshold used by other researchers. However, we believe that it is useful for descriptive purposes here given that more main meals were “excessive” in energy content than adhered to the public health recommendation of 600 kcal or less.

### Meaning of study

A sizeable proportion of main meals from fast food and full service restaurants were excessive in energy content, and we note that little or no information tended to be provided on menus that would allow consumers to identify menu options that were high in kilocalories versus those that were lower. Consumers tend to underestimate the number of kilocalories in large meals,^36^ ^37^ and this in combination with our findings makes recent calls to mandate energy labelling of restaurant food in the UK appropriate. Although such labelling is not common in UK restaurants, the best available evidence suggests that it is likely to have only a modest effect on consumers’ behaviour,^38^ so other public health measures to tackle energy intake out of the home will be needed. Because portion sizes of many food products have increased over time,^39^ ^40^ and reductions made to the portion size and energy density of food products are unlikely to be fully compensated for by consumers,^41^ ^42^ policy levers that result in the food industry reducing the number of kilocalories being sold to consumers are needed. This proposition is in line with the observation that changes to the food environment have played a key role in the emergence of the obesity problem, and measures are now needed to “renormalize” the food environment (for example, by downsizing food product portions).^43^

### Unanswered questions and future research

The reason why main meals in full service restaurants tended to be higher in energy than those in fast food restaurants is unclear, but multiple factors are likely to be involved, including the type of food sold. However, when we isolated our analyses to meal types that were routinely sold by both fast food and full service restaurants, we found that full service restaurant main meals still tended to be markedly higher in energy. Although these analyses were reduced in sample size, burger and chips main meals served in full service restaurants had a significantly higher energy content than those in fast food restaurants (414 kcal difference; P<0.001). Salad meals in full service restaurants also had higher energy content than in fast food restaurants, but the difference was not statistically significant (142 kcal; P=0.08). Thus, decisions about portion size, energy density of ingredients, and cooking methods are also likely to explain differences in meal energy content between full service and fast food restaurants. A further explanation is that the negative press received by the fast food sector because of poor nutritional quality of products may have caused restaurant chains in this sector to offer more lower energy meal options or reformulate existing products to reduce energy content,^24^ ^44^ whereas the full service restaurant sector does not seem to have experienced similar pressures. We also found marked variability between individual restaurants for energy content of main meals, and identifying why will be informative. In addition to the type of cuisine sold, individual restaurant market positioning and price range may be associated with energy content.

This study may be of use to future research efforts that examine whether major UK restaurant chains respond to public health calls to reduce the energy content their products. Likewise, if legislation is passed, the results of our study can be used to assess whether the introduction of energy labelling results in restaurant chains reformulating the nutritional content of meals, as seems to have been the case in the US.^45^ In this vein, characterising the nutritional quality of other parts of the UK food environment will be important, as this study did not examine nutritional quality of other market sectors (for example, coffee shops or online food ordering). Online services that allow consumers to have restaurant food delivered to their home are a recent development in the UK and will likely be increasing the number of meals consumed that are prepared out of the home.

### Conclusions

The energy content of a large number of main meals in major UK restaurant chains is excessive, and only a minority meet public health recommendations. Although the poor nutritional quality of meals from fast food restaurants has been well documented, the energy content of meals in full service restaurants in the UK tends to be higher and is a cause for concern.

**What is already known on this topic**

Eating out of the home is common in the UK
The poor nutritional quality of “fast food” has been well documented
The energy content of traditional “full service” restaurants has received less attention

**What this study adds**

The average energy content of main meals served in both fast food and full service restaurants in the UK is higher than public health recommendations
The proportion of main meals in UK restaurant chains that meet public health recommendations for energy content is smaller than the proportion that have an excessive energy content
Compared with fast food restaurants, full service restaurant meals in the UK contain significantly more kilocalories on average

## Web extra

Extra material supplied by authors

## Notes

Ethical approval: Not needed.

Data sharing: The final data sets containing restaurant meal descriptions and number of kilocalories for each restaurant used in analyses are available online at https://osf.io/cd597/.

Transparency: The lead author affirms that the manuscript is an honest, accurate, and transparent account of the study being reported; that no important aspects of the study have been omitted; and that any discrepancies from the study as planned (and, if relevant, registered) have been explained.
