# Supplementary material for: (Over)eating out at major UK restaurant chains: observational study of energy content of main meals
Source: BMJ. 2018 Dec 12;363:k4982. doi: 10.1136/bmj.k4982 (PMC6290483; doi:10.1136/bmj.k4982)
Supplement: Supplementary file 1 — Supplementary materials [file robe046276.ww1.pdf]

Supplementary materials for (over)eating out: observational study of energy content of main meals served in major UK restaurant chains

Supplementary table. Restaurant chains with ≥50 outlets identified and eligibility decisions

Restaurant chain name	N outlets	Eligible: Y/N (reason for ineligibility)
Subway	2559	Y
McDonalds	1249	Y
KFC	900	Y
Burger King	500	Y
Dixy Chicken	130	N (No calorie info)
Pizza Gogo	108	N (No calorie info)
Favourite Fried Chicken	88	N (No calorie info)
Five Guys	84	N (Individual items only)
Wimpy	78	Y
Yo! Sushi	77	N (Individual items only)
Pepe’s Piri piri	67	N (No calorie info)
Little Chef	61	N (No calorie info)
Chopstix	57	N (No calorie info)
Chicken Cottage	57	N (Individual items only)
Leon	51	Y
Wetherspoons	882	Y
Pizza express	490	Y
Nando's	360	Y
Frankie & Benny's	263	N (No calorie info)
Pizza Hut	261	Y
Hungry horse	261	Y
Prezzo	260	N (No calorie info)
Sizzling Pubs	231	Y
Harvester	204	Y
Vintage Inns	192	Y
Zizzi	174	Y
Toby Carvery	169	Y
Ember Inns	146	Y
Chef & Brewer	145	Y
Flaming Grill	131	Y
Wagamama	120	Y
Lounges	120	N (No calorie info)
Ask	114	Y
Carluccio's	103	N (No calorie info)
Old English Inns	100	Y
Café Rouge	95	N (No calorie info)
Côte	94	N (No calorie info)
Miller & Carter	93	N (No calorie info)
Gourmet Burger Kitchen	91	N (Individual items only)
Bella Italia	90	N (No calorie info)
Stone House	90	Y
TGI Fridays	82	N (No calorie info)
Bill's	82	Y
Chiquitos	73	N (No calorie info)
Slug and Lettuce	70	Y

Byron	69	N (No calorie info)
Table Table	69	Y
Pepe's Piri piri	67	N (No calorie info)
Wildwood Kitchen	56	N (No calorie info)
Las Iguanas	54	N (No calorie info)
All Bar One	52	Y
Giraffe	52	N (No calorie info)

Note. Reasons for ineligibility: 'No calorie info' = restaurant website did not provide calorie information for menu items, and did not provide when requested by email (n = 21), 'Individual items only' = restaurant only sold individual items on menus (e.g., sushi, burger and fries sold separately), not meals (n = 4).

Additional Information on Coding Instructions Used

We will only include meal options that are available all year round. We will therefore exclude seasonal menu options (e.g. meal options from a 'spring menu').

Menu options in which a customer can 'add' items to the meal (e.g. 'extra burger patty for £2') would not be included, as we are only including the default configuration of menu options offered by restaurants.

Food items that are sold individually and would typically be served as part of a meal are ineligible. For example, based on our eligibility criteria a burger on its own would not constitute a lunch or dinner meal in full, because in most restaurants serving burgers, burgers are served with at least one additional meal component (e.g. a burger and fries, a burger with a side salad). Further examples would be an individual hot dog or soup (e.g. with no bread), an individual sandwich (e.g. with no side of crisps, chips or salad garnish). Small plates 'tapas style' dishes are shared. Therefore, these menu options are not eligible.

Menu options that could be perceived to be 'individual items', but are typically served as a meal would be eligible. For example, pizza tends to be served on its own (e.g. no additional side), so a pizza would be classed as a main meal menu option.

A salad or a jacket potato that is clearly marketed as a side dish would not be eligible. However, based on our inclusion criteria, a multi-component salad or jacket potato that is marketed as a main meal (e.g. salad with halloumi) or jacket potato (e.g. jacket potato served with beans) would be eligible.

Main menu options that are labelled as 'healthy eating' or 'smaller appetites' and appear to be main meal options are eligible.

Carvery menu sections are meals that involve a portion of meat (or multiple portions of different meats) being served to a patron and the patron then selecting their accompanying sides (and size of portion) from a large list of menu options are not eligible (because it is not clear what constitutes a standard meal configuration).

If a restaurant has a specified take-away section of the menu that has menu options which are not available on the in-store eating menu, these menu options are not eligible.

Chi-Squared Results

Proportion of meals ≤600kcals from total sample: The Chi-squared test was significant ($\chi2(1) = 1762.1$, $p <.001$) demonstrating that full-service restaurants were less likely to offer meals exceeding public health recommendations (4% of all full-service meals) than fast-food restaurants (30% of all fast-food meals).

Proportion of meals ≥1000kcals from total sample: The Chi-squared test was significant ($\chi2(1) = 989.3$, $p <.001$) demonstrating that full-service restaurants (54% of all full-service meals) were more likely to offer extremely calorific meals than fast-food restaurants (20% of all fast-food meals).

Weighted Multi-Level Model

Because there was an unexpectedly large amount of variability in the number of meals that individual restaurants contributed to analyses we also conducted weighted multi-level analyses. We computed raw weights for level 1 (meal level) and level 2 units (restaurant level), using the formulas set out in Pillinger (2011). Conditional level 1 weights were calculated as 1 / wi|j, where wi|j was the probability of a meal being selected from all meals in the same restaurant. Level 2 weights were calculated as 1 / wj, where wj is the probability of selection of the restaurant that the meal belongs to from among all restaurants in the sample. When accounting for these weights in the model the two level structure (meals within restaurants) was a better fit of the data than a single level structure, $\chi2(1) = 4180$, $p < .001$, indicating that multi-level modelling was appropriate. The variance partition coefficient; the total residual variance which is attributable to restaurants rather than individual meals was 28%. Type of restaurant (full-service vs. fast-food) was a significant predictor, $\beta = 327$, SE = 61 (95% CIs 207 to 447), $p < .001$, explaining 45% of variance at the restaurant level. These results indicate that meals from full-service restaurants had 327kcals more energy than meals from fast-food restaurants, on average.

Mean kilocalorie content of specific meal types. Across all burger and fries/chips meals (N = 1,904; 1,010 full-service, 894 fast-food) the average number of kcals was 1171 (SD = 231). The weighted multilevel model demonstrated a two level structure was a better fit than a single level structure, $\chi2(1) = 688$, $p < .001$, and the variance partition coefficient was 36%. Type of restaurant (full-service vs. fast-food) was a significant predictor, $\beta = 279$, SE = 109 (95% CIs 66 to 492), $p < .001$, explaining 13% of variance at the restaurant level and indicating that burger meals in full-service restaurants had 279kcals more energy than in fast-food restaurants, on average. Across salad meals (N = 304; 92 full-service, 212 fast-food) the average number of kcals was 446 (SD = 182). A two level structure was a better fit than a single level structure, $\chi2(1) = 275$, $p < .001$, and the variance partition coefficient was 78%. Type of restaurant (full-service vs. fast-food) explained 8% of variance at the restaurant level and full-service restaurant salad meals had on average 201kcals more than fast-food meals, although restaurant type was not a statistically significant predictor in the model, $\beta = 201$, SE = 128 (95% CIs -49 to 451), $p = .058$.
